# Dynamics and Structural Responses to Cis–Trans Isomerization in Bacterial Lipid Bilayers

**DOI:** 10.1021/acsomega.5c04983

**Published:** 2026-01-01

**Authors:** Saad Raza, Troy H. Sievertsen, Majid Jafari, Josh V. Vermaas

**Affiliations:** † Plant Research Laboratory, 3078 Michigan State University, 612 Wilson Road, East Lansing, Michigan 48824, United States; ‡ Department of Biochemistry and Molecular Biology, Michigan State University, 609 Wilson Road, East Lansing, Michigan 48824, United States

## Abstract

Bacterial and eukaryotic cells must respond to a changing environment and have multiple adaptive mechanisms to respond to environmental stresses. Exogenous stresses, such as temperature fluctuations and osmotic pressure, are known to influence cell membrane fluidity and gene expression. To maintain membrane homeostasis, Gram-negative bacteria show a short-term membrane composition response to temperature changes. Specifically, these bacteria isomerize unsaturated fatty acid tails in their bilayers, switching unsaturation sites from the more common cis isomer to the trans isomer. Cis–trans isomerization in unsaturated fatty acids increases cell membrane rigidity, decreasing the fluidity of the lipid acyl tails. These changes maintain membrane homeostasis, but the effect size is difficult to quantify *in vivo*. In this work, we explore the impact of fatty acid cis–trans isomerization on the properties and dynamics in a membrane model based on *Pseudomonas putida* using molecular dynamics (MD) simulation. In our hypothetical model, we convert between all-cis and all-trans membranes and report on the variation in membrane properties under these conditions. In addition to changes in membrane thickness and lipid diffusion, we find that the unsaturation site for a cis fatty acid has a higher probability of coming to the membrane surface than the equivalent trans fatty acid. The reduced availability of unsaturation sites on the membrane surface may have downstream implications for their accessibility to enzymatic attack, potentially influencing the activity of cis–trans isomerase and other peripheral membrane proteins that act on lipid unsaturations. Since cis–trans isomerization can occur rapidly without new lipid biosynthesis, natural selection has adopted cis–trans isomerization as one of many responses to environmental stress.

## Graphical abstract

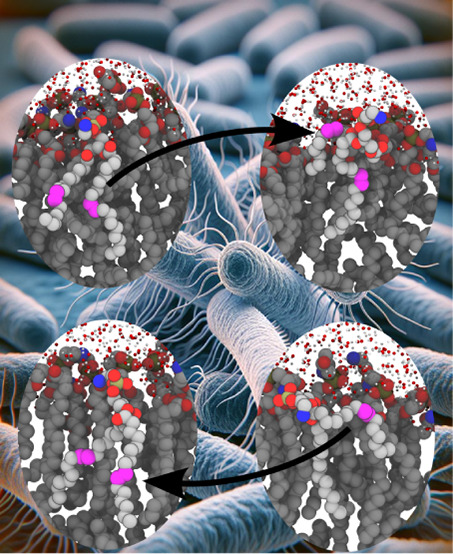

## Introduction

Living cells have evolved various mechanisms to acclimate to environmental changes. The ability of an organism to become accustomed to new ambient conditions plays a vital role in keeping cells alive. Bacterial cells employ multiple adaptive mechanisms under various environmental stresses.^1,2^ For example, heat shock and osmotic pressure influence eukaryotic cell membrane fluidity and gene expression.^3^ Bacteria respond to environmental conditions through both short- and long-term adaptations. Short-term responses need to occur immediately to avoid lipid phase transitions due to temperature fluctuation, osmotic stress, or toxic substances.^4,5^ Gram-negative bacteria are known to show a short-term response to environmental stress by isomerizing unsaturated cis lipid tails to trans through the cis–trans isomerase (Cti) enzyme.^5–9^ This alteration causes increased packaging of acyl chains in trans conformation, which increases the rigidity of the phospholipid membrane and leads to an increase in membrane stability under stress.^9–11^

*Pseudomonas putida*, a Gram-negative bacterium, is resistant to harsh conditions like high pH or nutrient scarcity.^12,13^ Due to its fast growth in simple bacterium culture, *P. putida* has drawn researchers’ attention to its use in the industry, from synthesizing biopolymers to degrading xenobiotic substances.^14^ For instance, polyhydroxyalkanoates (PHA), a biocompatible polymer produced by *P. putida*, have various applications in biodegradable packaging and tissue engineering,^12^ and *P. putida* can incorporate multiple exogenous aromatics into its metabolism for industrial use.^15–18^ Besides polymer production, this bacterial strain is an excellent host for expressing genes from other bacteria. As an example, *P. putida* can produce some complex natural products of myxobacteria, which are applied as high-value pharmaceuticals.^19,20^ The tractability for engineering *P. putida* makes it an excellent candidate for a starting point to investigate cis–trans isomerization,^21–23^ particularly as cis–trans isomerization is heavily regulated in *Pseudomonas* species,^24^ which may be exploited to change membrane properties by design.

Determining the degree of isomerization at the benchtop while simultaneously measuring the membrane properties is exceptionally difficult. Prior studies have attempted to quantify the isomerization rate and degree of isomerization experimentally and have identified a low overall yield of isomerized lipids of between 0.5 and 4%.^25^ Trans lipids are observed only quickly after stress is introduced to the bacterial culture,^26–30^ which is faster than other stress responses like increasing cardiolipin content, which can take hours to be effective in bacteria.^30–33^ Using the freedom afforded by computational approaches, we can go beyond what is physiologically observed and pose the question of what impact isomerization has on membrane dynamics and structures by comparing all-cis and all-trans membranes in molecular dynamics (MD) simulations. By studying isomerization in this well-controlled environment, we are well-positioned to offer insight into well-controlled and precise interrogations into the molecular motions of lipids and proteins at the atomic scale.^34–36^ Previous simulation studies into cis/trans isomerization had identified trans-specific interactions with cholesterol.^37^ Rather than repeating those studies on model membranes, the *P. putida* membrane offers a greater diversity of lipid types and unsaturation profiles than prior studies.^38,39^ Previous studies have shown that isomerization reduces the membrane fluidity under environmental stress like antibiotics or organic solvents, thereby enhancing bacterial resistance to these stressors.^4,5^ The membrane fluidity can also influence the water dynamics at the interfacial region.^40,41^ The membrane fluidity is derived from the nanoscale structure of the membrane, and so membrane dynamical and structural properties like membrane thickness, area per lipid, lipid order, and diffusivity are expected to be different. By comparing the membrane structure and dynamics between membranes composed of all-cis and all-trans fatty acids, we quantify the triggered response to environmental stresses in *P. putida* noted in prior LC/MS experiments,^39^ albeit in a more exaggerated condition that allows for robust conclusions over accessible simulation time scales. We find that all-trans membranes are thicker with a lower area per lipid and diffuse noticeably slower than the all-cis membrane controls. Crucially, the cis unsaturation sites are more exposed to solution and may be the origin for the increased bioavailability of cis fatty acids in addition to altering the membrane mechanical properties.^11,42^

## Methods

### Modeling *Pseudomonas putida* Membrane

*Pseudomonas putida* is the model organism we are most interested in, and detailed lipidomics is available in the literature.^39^ The membrane headgroup composition was 12:7:1 for Phosphatidylethanolamine (PE):Phosphatidylglycerol (PG):Cardiolipin (CL). No sterols were added, as the lipidomics data in the wider literature has not found sterols in *Pseudomonas putida* membranes, but only phospholipids and lipid A in the outer membrane.^43^ The membrane patch used in this study was only 100 lipids, which is sufficient to measure membrane structural properties such as thickness, area per lipid, lipid order, and so on while reducing the overall simulation cost. The detailed composition of the lipids along with tails is given in Table 1 (Figure 1A), and results in membrane systems with approximately 30,000 atoms. The membrane was generated using CHARMM-GUI^44^ (Figure 1B). The initially generated membrane has unsaturated acyl tails in the cis conformation, which were converted into all-trans by rotating the dihedral angle for the double bond by 180° using in-house Tcl scripts (Figure 1C). Five replicates of cis and trans membrane configurations were generated for running MD simulations.

**Figure 1:**
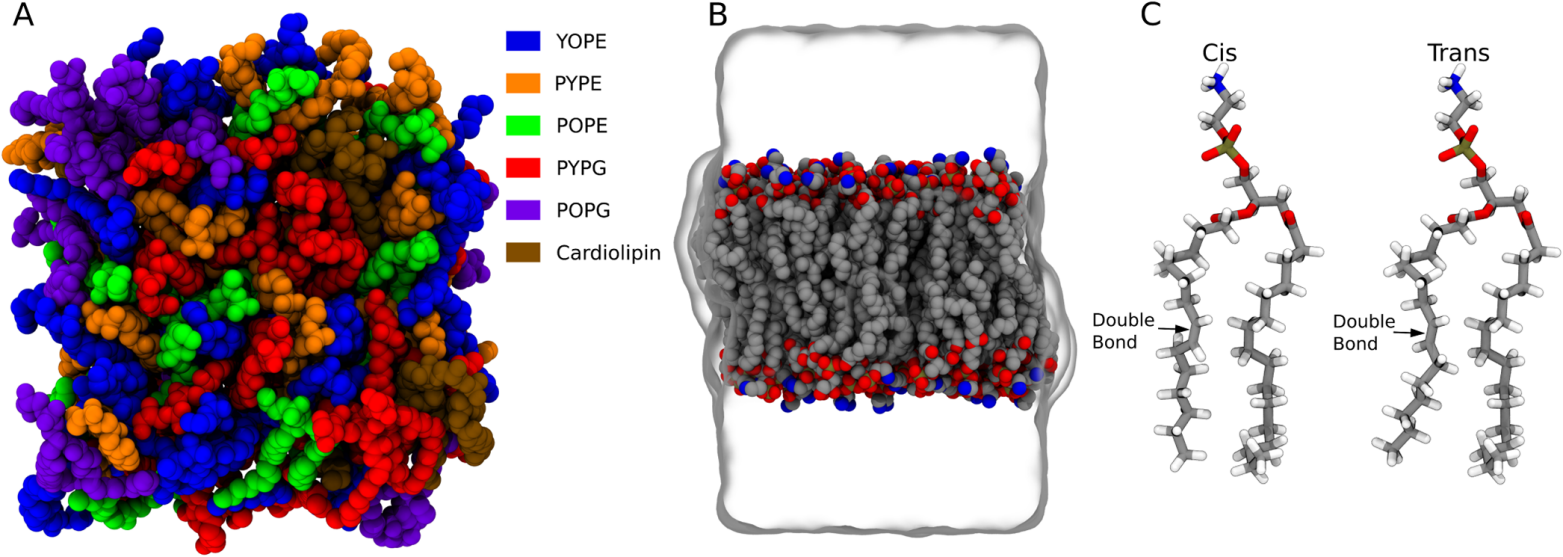
(A) Lipid headgroup distribution of one leaflet is shown above. Heavy atoms of the lipids are represented as van der Waals beads. Each lipid residue is color-coded according to the legend. (B) Molecular dynamics simulation system with water represented as a surface representation and heavy atoms of the lipids as van der Waals beads. Here, red represents oxygen, blue nitrogen, gray carbon, and light brown phosphorus. (C) Cis and trans conformations of the lipid PYPE are given in licorice representation. The color representations are the same as in (B).

**Table 1:** Head Group and Tail Composition of One Leaflet of the *Pseudomonas aeruginosa* Bilayer Used for MD Simulation^a^

Lipid Type	Total Number	Full Name	Residue Name	Tails
PE(32:1)	10	1-palmitoyl-2-palmitoleoyl-*sn*-glycero-3-PE	PYPE	16:0/16:1
PE(34:1)	7	1-palmitoyl-2-oleoyl-*sn*-glycero-3-PE	POPE	16:0/18:1
PE(34:2)	14	1-palmitoleoyl-2-oleoyl-glycero-3-PE	YOPE	16:1/18:1
PG(32:1)	11	1-palmitoyl-2-palmitoleoyl-*sn*-glycero-3-PG	PYPG	16:0/16:1
PG(34:1)	6	1-palmitoyl-2-oleoyl-*sn*-glycero-3-PG	POPG	16:0/18:1
CL(32:0,36:2)	2	1′[1,2-dipalmitoyl-*sn*-glycero-3-phospho],3′[1,2-dioleoyl-*sn*-glycero-3-phospho]-bis glycerol	PVCL2	16:0/16:0,18:1/18:1
Sum	50			

a The 2D structures are given in supplementary figure S1.

### Simulation Protocol

We take the unusual step of running two sets of long equilibrium simulations, both in NAMD and in GROMACS. The original set was run with NAMD; however, we also ran simulations in GROMACS to compare and contrast, leading to two sets of simulations to compare results against. To our surprise, there were substantial differences between both simulation engines, even as we took care to make reasonable simulation choices for a CHARMM36 lipid simulation.^45^ Thus, we analyze both trajectories and comment on their differences in the appropriate places.

#### NAMD Equilibrium Simulation

The original NAMD simulations were done using the CHARMM36 force field for lipids.^45^ Membrane models were simulated in TIP3P water models with a 12 Å nonbonded cutoff.^46^ Anisotropic pressure coupling was held consistent using the Langevin piston method at a pressure of 1 atm.^47^ Using the SETTLE algorithm to fix hydrogen bonds at the same length, the time step was at 2 fs.^48^ Temperature was controlled with a Langevin thermostat at 310 K with 1 ps^–1^ damping. Long-range electrostatic interactions were calculated with a particle mesh Ewald (PME) grid with 1.2 Å spacing.^49,50^ LJ interactions were smoothed over the range of 10–12 Å.^51^ LJ correction was applied to improve energy conservation during switching.^52^ The energy was minimized in 1000 steps using NAMD version 2.14.^53^ The system was then allowed to equilibrate for 50 ps in the NVT ensemble, and afterward it was allowed to equilibrate in the NPT ensemble for 10 ns with a margin of 10 to allow simulation box adjustment. Production simulations were carried out on NAMD version 3.0alpha9 for 1000 ns with the default margin.^54^

#### GROMACS Equilibrium Simulation

The equivalent GROMACS simulation protocol was performed using GROMACS 2022.3 (GROningen MAchine for Chemical Simulations).^55^ To remove the steric clashes of the system, energy minimization was done using the steepest descent algorithm for 5000 steps with an energy threshold of 1000 kJ mol^–1^ nm^–1^. The CHARMM36 force field for lipids was used for running the MD simulation.^45^ The system was heated using the NPT ensemble for 1 ns. Temperature was set to 310 K and was controlled with a Nosé–Hoover thermostat.^56–58^ LINCS was used to constrain the hydrogen movement.^59^ The time step was set for 2 fs. Electrostatic interactions were calculated using the particle mesh Ewald algorithm with a 12 Å cutoff.^49^ For a long-range van der Waals interaction, a 12 Å cutoff was used. Semi-isotropic pressure control was applied using the C-rescale method implemented in GROMACS.^60^ The system was equilibrated for 1 ns using the same protocol. The production was run for 1 μs. After every 10 ps, coordinates and velocities were written in the file.

### Analysis

Structure files and MD simulation trajectories were visualized and analyzed using Python-enabled VMD 1.9.4a58.^61^ VMD provides a Python interface to utilize the NumPy numerical library and plotting tools like matplotlib.^62,63^ To study the structural difference of the membrane in cis and trans conformations, membrane structure measurements like membrane thickness, area per lipid, membrane diffusion, and membrane order parameters were calculated from the MD trajectory. The membrane thickness was calculated as the distance between the phosphate groups of the two leaflets, and the volume was determined by multiplying the *X* and *Y* dimensions of the simulation box by the membrane thickness. The relative *z* distribution of double bonds and phosphate atoms was calculated using the mean position of phosphate atoms at each frame to define a reference plane and then measuring the atomic positions for either the carbons involved in a double bond or the phosphorus and oxygens of the phosphate groups relative to that plane.

Determining the area per lipid for individual residues is calculated in two steps. We first determine the size of the membrane in the *x*–*y* plane and then subdivide the membrane plane into a grid with a maximum of 1 Å between grid points. We then lay out this grid at *z* = −18 and *z* = 18 Å from the membrane midplane and ask, through spatial search methods in Scipy,^64^ what lipid residue is nearest to each grid point. Since we know how many of each lipid are present and we know the area represented by each square in the grid, we can determine the area per lipid for each specific residue type in the simulation.

Membrane order parameter was determined using the MEMBPLUGIN for VMD.^65^ The order parameter is calculated using the equation:

1 $$S_{CH}=\left\langle 3\cos^{2}\theta -1\right\rangle /2$$

*S*_*CH*_ is the lipid order parameter calculated, and angle θ is between the C–H bond vector and the bilayer normal.

The membrane diffusion coefficient (*D*) for lipid residues is calculated from the mean squared displacement of lipid residues (MSD):

2 $$MSD=\left\langle \right|x-x_{0}{|}^{2}\rangle$$

3 $$D=\frac{MSD}{2nt}$$

*x*_0_ is the reference position of the lipid residue, and *t* is the total simulation time. For membrane diffusion calculations, lipid displacement is only in the *X* and *Y* dimensions, and so we consider only *n* = 2 degrees of freedom.

## Results and Discussion

Prior reports have indicated that cis and trans lipids yield noticeable changes in lipid packing and nanoscale structure.^10,37^ Lipid packing in turn alters membrane properties like membrane fluidity, a key regulator of bacterial osmotic pressure and chemostasis.^66,67^ We use MD simulation to visualize and quantify the structural and mechanical changes induced by cis–trans isomerization within a *Pseudomonas* membrane.

### Structural Comparison between all-*cis* and all-*trans* Membranes

Multiple structural properties are accessible via MD simulation through direct observation, such as membrane thickness, surface area per lipid, and total membrane volume. The membrane geometry, quantified by membrane thickness and surface area per lipid, is influenced by membrane saturation and isomerization state and governs the membrane fluidity as the organism responds to stress.^68^ Concretely, membrane thickness is critical for proper protein function, as mismatched hydrophobic thicknesses and thus membrane compressibilities can dramatically lower transport activity.^69,70^ Lower membrane thicknesses can increase membrane fluidity and thus permeability as the membrane exchanges between ordered and disordered states.^68,71,72^ Membrane fluidity can also be determined from structure based on the lipid order parameter as well,^73^ which is why it is such a key metric for assessing membrane structure. We will quantify all of these properties in turn from our MD simulations.

We quantify the membrane thickness by tracking the distance from phosphate to phosphate between the two equivalent membrane leaflets. The all-*cis* membrane showed a lower thickness compared to the all-*trans* membrane (Figure 2A), and the all-*cis* membrane has a larger area per lipid (Figure 3) in both NAMD and GROMACS simulations. The trade-off between thickness and area is a known phenomenon from prior X-ray scattering experiments.^74^ Indeed, the area per lipid decreases by approximately 5–8 Å^2^ for most trans phospholipids compared with their cis counterparts, which lines up with prior simulations of less complicated membranes.^11^ The exception to this is cardiolipin, which has a much smaller change in surface area than the other lipids, both in absolute terms and on a percentage basis, potentially as a result of better packing between the four fatty acid tails in native cardiolipin.

**Figure 2:**
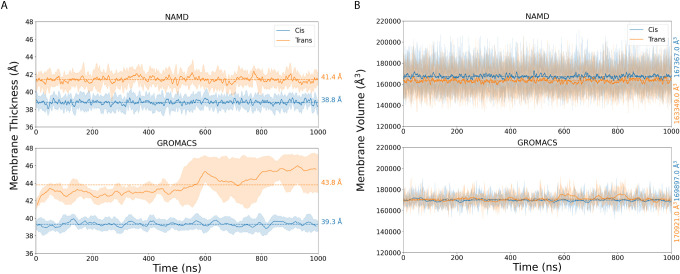
(A) Membrane thickness calculated from the phosphate-to-phosphate distance from opposite leaflets for cis and trans membranes. (B) Membrane volume calculated for cis and trans membranes. In both graphs, the distribution over all five simulation replicates is represented as a shaded region. The solid line is the timeseries for the mean thickness or volume across all five simulation replicates. The mean value across all collected data is shown as a dashed line, with the values reported on the right-hand edge. As shown in the legend, the all-cis membrane traces are drawn in blue, while the all-trans membrane traces are drawn in orange. The upper panel results are from NAMD simulations, and the lower panel results are from GROMACS simulations.

**Figure 3:**
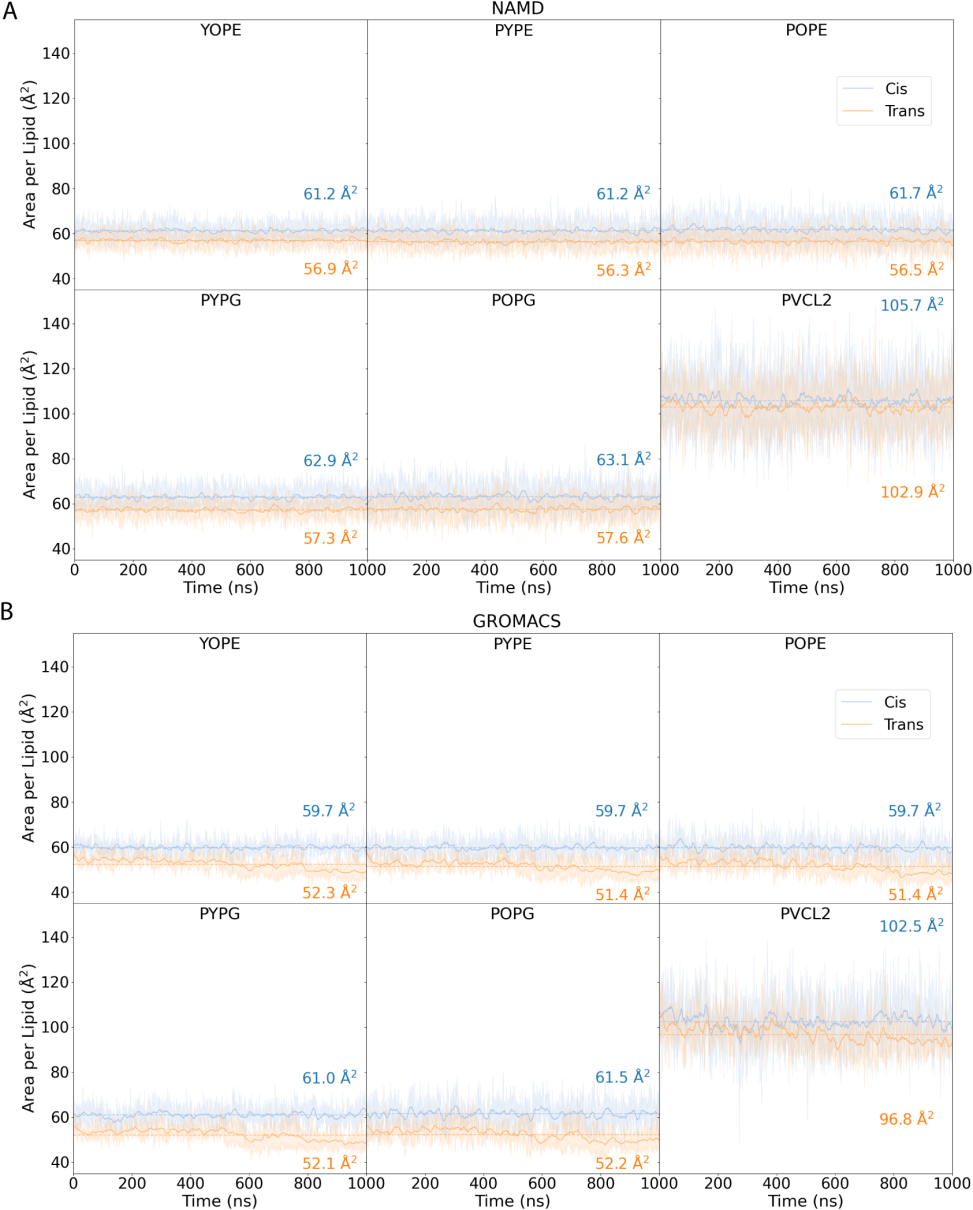
Area per lipid for six different lipid residues in all-*cis* and all-*trans* lipid bilayers in (A) NAMD and (B) GROMACS simulations. The names of each lipid are given in Table 1. In all these graphs, the overall distribution for the area per lipid observed over simulation replicates is represented as a shaded area around the mean value, which is represented as a solid line. The mean over all data is shown using a dashed line, with the mean value reported on the right-hand edge of the graph.

Prior studies often mention lipid packing defects caused by cis lipid acyl tails, particularly when compared with trans acyl tails.^11,24^ The opposing trends between thickness and area per lipid do not make it obvious how the packing is changing. By quantifying the total membrane volume, taken to be the membrane surface area multiplied by the membrane thickness, we find that the total volume is larger for the all-*cis* membrane, indicating tighter packing and a greater overall density for trans lipids (Figure 2B) in both simulations. Experiments have also indicated that trans lipids have tighter packing and are intermediate between typical cis lipids and unsaturated acyl tails.^74–76^ However, by quantifying the volume change, we see that the change in packing efficiency is not all that large, with a 2.5% change in total membrane volume. The increased packing density and ordering upon isomerization to trans is anticipated to have far-reaching and often deleterious effects, such as changing membrane permeability by potentially orders of magnitude,^77,78^ so isomerization is truly a fast response to maintain membrane homeostasis.

Continuing our exploration of the membrane structure, we notice more commonalities with previous reports. The double bonds at the unsaturation sites introduce a kink in the lipid tail, particularly for cis stereoisomers. By measuring the angle made between the terminal carbon, the kink, and the carbonyl carbon, we note that the kink is around 15° greater in magnitude for cis lipids compared with trans (Figure S2A) in NAMD simulations, analogous to prior reports.^11^ This kink increases to 25° in magnitude for cis lipids compared to trans (Figure S2B) in GROMACS simulations. This manifests with less ordered unsaturated cis fatty acids compared with trans fatty acids, both for typical phospholipids (Figure S3) as well as cardiolipin (Figure S4). The greater order for trans fatty acids changes the phase behavior for membranes in general^79^ and is associated with transitions from a disordered L_*d*_ to a more ordered L_*o*_ or L_β_ phase, with all the accompanying changes in membrane properties.^72,78^ The higher fluctuation in the lipid order of the trans membrane in GROMACS simulations is due to the change in phase during the simulation (Figures S3B and S4B).

From different properties calculated for the trans membrane, the trans membrane is stiffer in GROMACS compared to the NAMD trajectory (Figure 4). After 475 ns of simulation, the trans membrane transitions to a more ordered arrangement. The phase transition to a liquid-ordered phase is irreversible along the remaining 525 ns of simulation. This transition of the trans membrane into a highly ordered membrane is only seen in GROMACS, and its origins are not clear. We are reasonably confident that GROMACS is handling the CHARMM36 force field correctly, and we can see no reason why the thermostat or barostat choices would have such a clear change. At this stage, we cannot be certain which molecular simulation engine is correct for such a strange all-trans system, and this warrants more exploration beyond the scope of this study.

**Figure 4:**
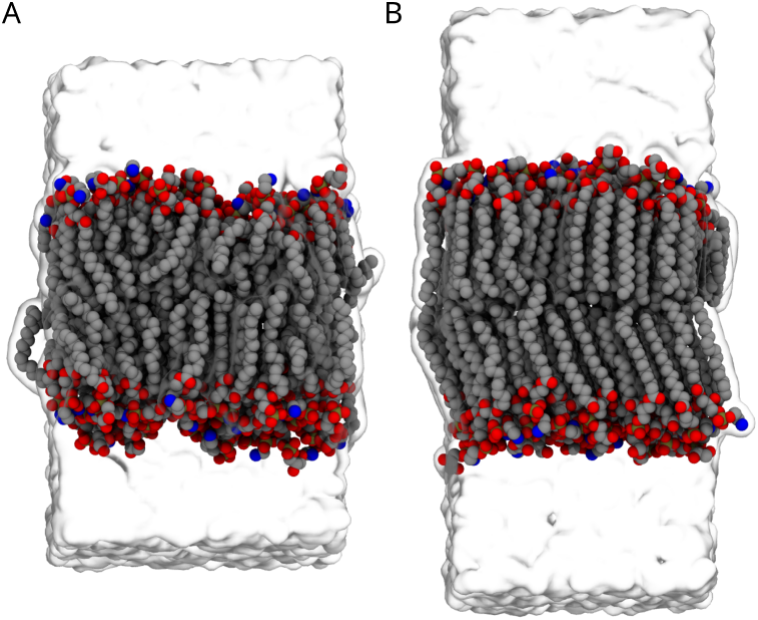
Phase change of lipids in the transmembrane during GROMACS simulations. Snapshot of MD taken at the start of the MD simulation (A) and at the end of the simulation (B). At the end of the simulation, the membrane is more ordered and in a crystal-like state. Water is represented as surface representation, and heavy atoms of the lipids as van der Waals beads.

### Membrane Diffusion

The lipid structures characterized above are similar to what has been found previously and are broadly in line with the literature consensus despite the greater complexity of lipid composition. A key question is whether the membrane dynamics are similarly replicated across different lipid types. From Figure 5A, it is clear that the mean squared displacement is larger for cis lipids than for trans lipids, and so we should expect the higher diffusion for the disordered all-*cis* membranes in NAMD. This same pattern is seen in the GROMACS simulation but with reduced displacement compared to the NAMD (Figure 5C). Lipid lateral diffusion is a readily accessible metric that can be compared to both experiments and varying simulation models^80,81^ with a wide range of characterized values depending on the membrane. Some report self-diffusion coefficients from simulation of around 0.06 × 10^–8^ cm^2^,^82^ while others report substantially higher diffusion coefficients of 3–8 × 10^–8^ cm^2^/s,^83,84^ although the membrane size in simulated bilayers can dramatically change the measured lipid lateral diffusion.^84,85^ In our simulations, we observe diffusion on the higher end of this range (Figure 5B), in line with current expectations for a membrane in the L_*d*_ phase run with a small number of lipids.^84^

**Figure 5:**
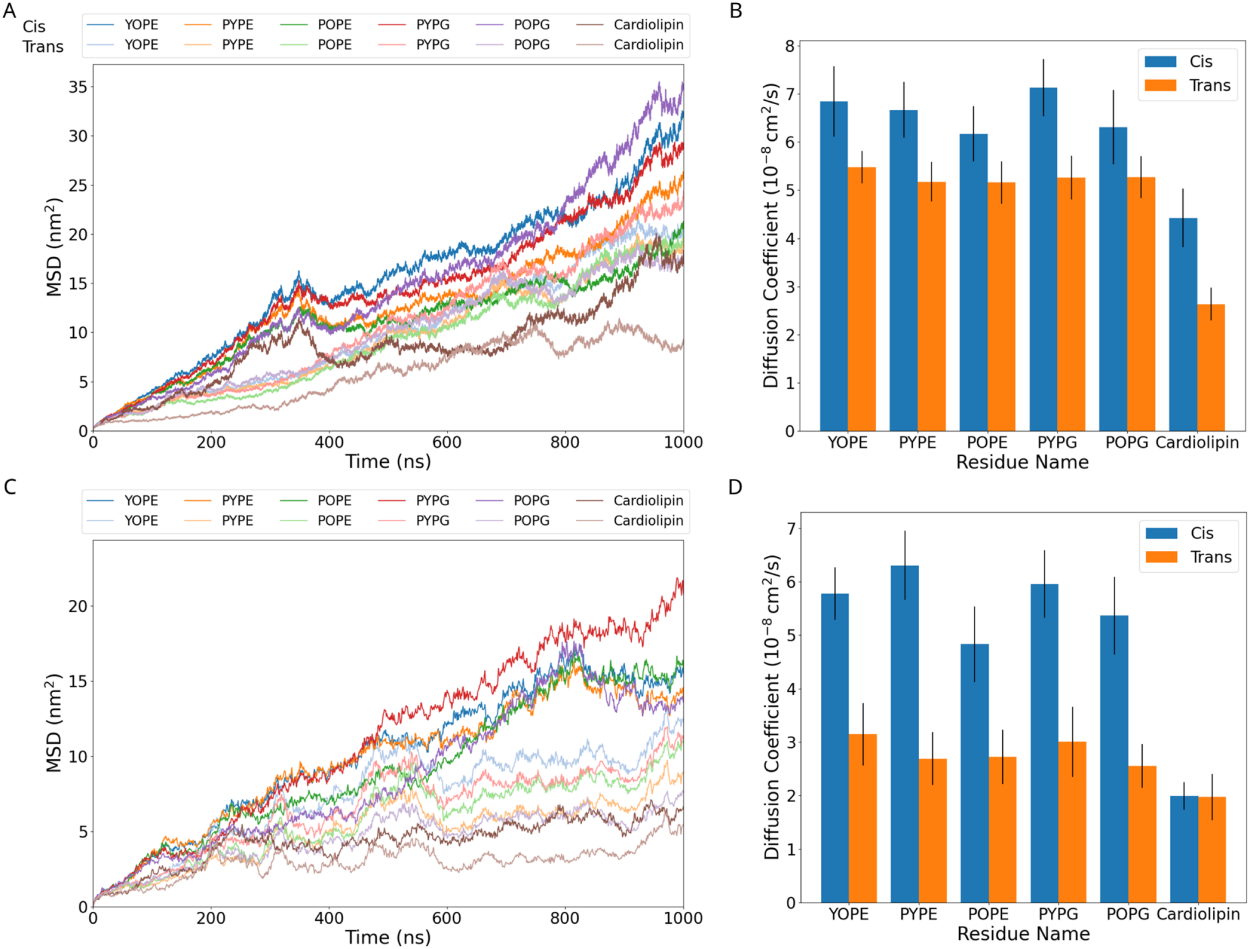
(A,C) Mean square displacement of lipid residues in cis and trans membranes relative to time zero by eq 2. (B,D) Average diffusion coefficient of lipid residues in the cis and trans membranes calculated by eq 3 over the simulation trajectory, dividing each trajectory into 200 ns chunks for independent analysis. The results for NAMD are on the top panel (A,B), and the bottom panel are the results from GROMACS (C,D). The error bars for the diffusion coefficient represent the standard error, considering both the standard deviation and the independent samples (25 for NAMD and 15 for GROMACS). Values of the diffusion coefficient are given in supplementary table S1.

While we know that our diffusion coefficients are likely overestimated due to the small size of the simulation box,^84^ we were anticipating that specific phospholipids may diffuse more quickly or slowly based on the structure of the individual acyl tails. However, we instead see that the diffusion coefficients between phospholipids are highly similar, with the diffusion coefficients for cis phospholipids ranging between 6 and 7 × 10^–8^ cm^2^/s, and trans phospholipids demonstrating diffusion coefficients between 5 and 5.5 × 10^–8^ cm^2^/s (Figure 5B) in NAMD simulation. GROMACS simulation shows 0.3 times slower for the cis membrane and 0.5 times lower for trans compared to the diffusion in NAMD (Figure 5D). The exception is the much more massive cardiolipin that diffuses considerably more slowly than the other lipids. The impact of isomerization on diffusion, approximately a 10–20% decrease for trans membranes, is much smaller than other modifications bacteria make to their membranes, such as glycosylation in lipopolysaccharides, which may slow down lipid lateral diffusion by 100-fold.^86,87^

### Lipid Tail Surface Accessibility

In our preliminary visual trajectory analysis, we were readily able to find cis double bonds near the membrane surface (Figure 6B). By contrast, trans double bonds were rarely observed at the membrane surface. To quantify this observation, we want to measure how often double bonds are near the surface (Figure 6A). Since the membranes have different thicknesses, we do not measure from the membrane midplane in Figure 6A, but rather measure the position of the double bonds relative to the phosphate groups. We find in the NAMD simulation (Figure 6A) that the cis double bonds are on average closer to the membrane surface than the trans double bonds. While the position of the maximum probability shifts by less than 2 Å, the asymmetric distribution for atoms in cis double bonds places them occasionally very close to the membrane surface. In the NAMD simulation, the population within 5 Å of the membrane phosphate plane is 0.8% in cis membranes but only 0.15% in trans membranes. The cis lipids’ double bonds are thus five times more likely to appear near the membrane surface compared to the trans lipids in NAMD simulations.

**Figure 6:**
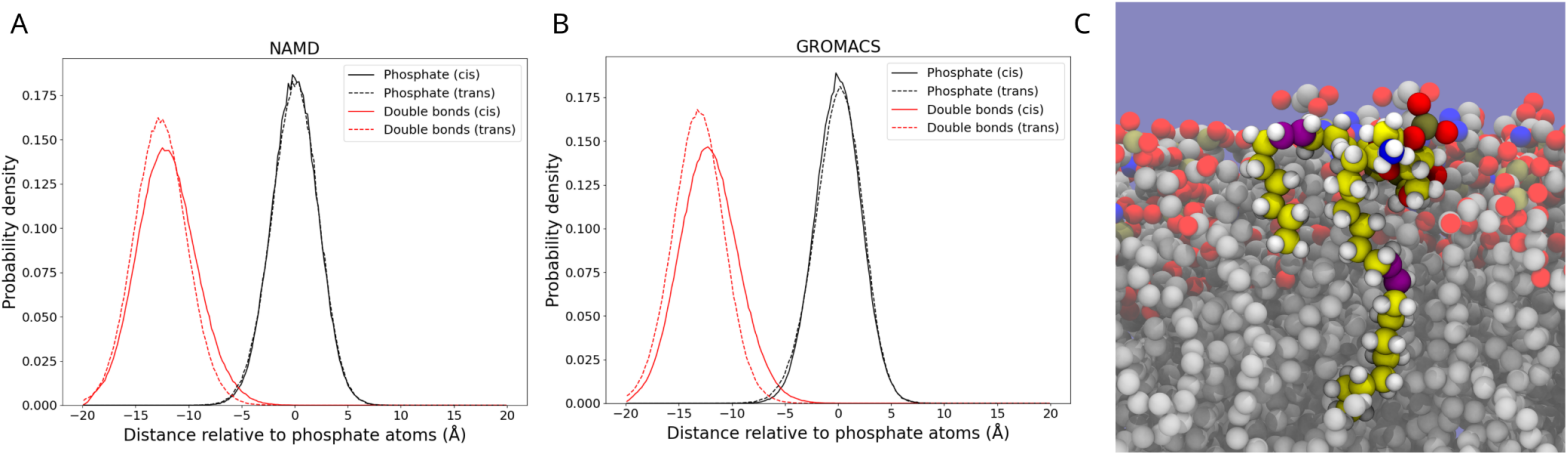
Relative probability distribution of double bonds and phosphate atoms relative to the membrane surface computed by the mean position of all phosphate atoms in (A) NAMD simulations and (B) GROMACS simulations. (C) Example snapshot highlighting a cis double bond (purple atoms) at the membrane surface. To better visualize the membrane, hydrogen atoms (white) are only drawn on the highlighted lipid, with the remaining carbons of the highlighted lipid shown in yellow. The remainder of the membrane context uses gray carbon atoms, red oxygen atoms, brown phosphorus atoms, and blue nitrogen atoms. The background water molecules are represented by the solid blue surface and are not represented for visual clarity.

The cis lipid in GROMACS simulation (Figure 6B) shows a similar pattern of double bond distribution relative to that of phosphate atoms. The trans lipids are more rigid in GROMACS simulation and hardly ever come to the membrane surface compared to the simulation results from NAMD. In GROMACS simulation, the population within 5 Å of the membrane phosphate plane is 0.6% in cis membranes, but only 0.05% in trans membranes. The probability of catching the lipid on the surface in GROMACS is still lower compared to the NAMD simulation, but relative to the trans membrane in the GROMACS simulation, the ratio is 11 times higher.

The increased surface accessibility for cis lipids compared with trans lipids is likely a boon for peripheral membrane proteins that act on membrane lipids, such as Cti. Our results indicate that cis lipids would readily present their double bonds to the membrane surface, where they would be accessible to subsequent protein activity, including to make natural products.^88^ By contrast, the relatively inaccessible trans acyl tails would most likely need to be recognized by transmembrane proteins. Thus, the enzymatic chemistry of trans acyl tails would likely be slower, as peripheral membrane proteins are smaller and thus would diffuse faster than larger transmembrane proteins would.

## Conclusion

*Pseudomonas putida* can live in a wide range of habitats.^89–92^ The natural habitat for microorganisms can be harsh and unforgiving.^93^ Through natural selection and evolution, these bacteria have adopted multiple mechanisms to survive in harsh or stressful conditions.^26–33^ One mechanism that we have interrogated here is the cis–trans isomerization of lipids in the membrane bilayer,^5,26^ asking the question of what would happen for a fully isomerized membrane. This cis–trans isomerization is hypothesized to change the fluidity of the membrane to protect the membrane from denaturation under stress conditions.^6^ We quantified the fluidity of the membrane in terms of different physical properties like membrane thickness, lipid order parameter, and diffusion coefficient. All of these parameters point toward the stiffening of the membrane by conversion of cis lipids to trans lipids, as all-trans membranes are thicker with greater order and diffuse more slowly. The rigidity in the membrane and increased surface tension allow a quick response to the external stress, prior to slower responses that involve novel lipid synthesis accounting for a greater response.^26^

What we find particularly striking is that the distribution of the double bonds relative to the surface of the membrane in cis and trans also explains the evolutionary need for natural lipids to be in the cis configuration in the first place. Double bonds in the cis region are approximately 5× more accessible to the membrane surface than the equivalent trans double bonds, which makes it easier for peripheral membrane proteins (such as Cti) to act on them. Cis fatty acids are thus more responsive to processing and metabolism than trans fatty acids, which could underlie the mechanism for why trans fats are such a risk factor in human health^94–96^ and are so hard to remove biologically. Beyond the mechanical effect of trans lipids to stiffen the membrane, their low availability to enzymatic action compared to cis fatty acids would decrease their chances of being metabolized, thereby lingering longer in membranes.

To us, another clear conclusion is that there are clear changes in the membrane simulation behavior between NAMD and GROMACS. We tried to keep most of the parameters equivalent. The difference that we see may or may not be due to the difference in simulation parameters, which are slightly different in each simulation engine.^97^ But a phase change is a very drastic shift in dynamics, which may or may not be linked to these factors, and it is up for debate for future discussion. For what should ostensibly be the same force field and input structures, seeing such large structural and dynamic changes is unexpected for a well-equilibrated system. We are unsure which simulation engine is correct but take pains to emphasize that they do agree on the general findings outlined above. These findings warrant further exploration through experiments and revisiting the computational methodologies, which is beyond the scope of this article.

## Supplementary Material



## Data Availability

We have deposited the reduced directory structure to support all of our conclusions in Zenodo (10.5281/zenodo.14963106).

## Notes

Figure S1 shows the 2D structures of the lipids used in the composition of the lipid membrane; Figure S2 quantifies the angle made at the unsaturation for cis vs trans unsaturation sites; Figures S3 and S4 measure the membrane ordering for the different lipids present in the simulation box (PDF)

## §

Department of Chemistry and Biochemistry, University of Delaware, Newark, DE 19716

## ∥

Department of Biochemistry, Purdue University, 175 South University St., West Lafayette, IN 47907.
