# Supplementary material for: Dynamics and Structural Responses to Cis–Trans Isomerization in Bacterial Lipid Bilayers
Source: ACS Omega. 2026 Jan 1;11(1):449–59. doi: 10.1021/acsomega.5c04983 (PMC12809570; doi:10.1021/acsomega.5c04983)
Supplement: Supplementary file 1 [file ao5c04983_si_001.pdf]

Supporting Information: Dynamics and structural responses to cis-trans isomerization in bacterial lipid bilayers

Saad Raza,[†,¶] Troy H. Sievertsen,[‡,§] Majid Jafari,[‡] and Josh V. Vermaas*,[†,‡]

†*Plant Research Laboratory, Michigan State University, 612 Wilson Road, East Lansing MI 48824*

‡*Department of Biochemistry and Molecular Biology, Michigan State University, 609 Wilson Road, East Lansing MI 48824, East Lansing MI 48824*

¶*Current address: Department of Chemistry and Biochemistry, University of Delaware, Newark, DE 19716*

§*Current address: Department of Biochemistry, Purdue University, 175 South University St., West Lafayette, IN 47907*

E-mail: vermaasj@msu.edu

Phone: +1 (517) 884-6937

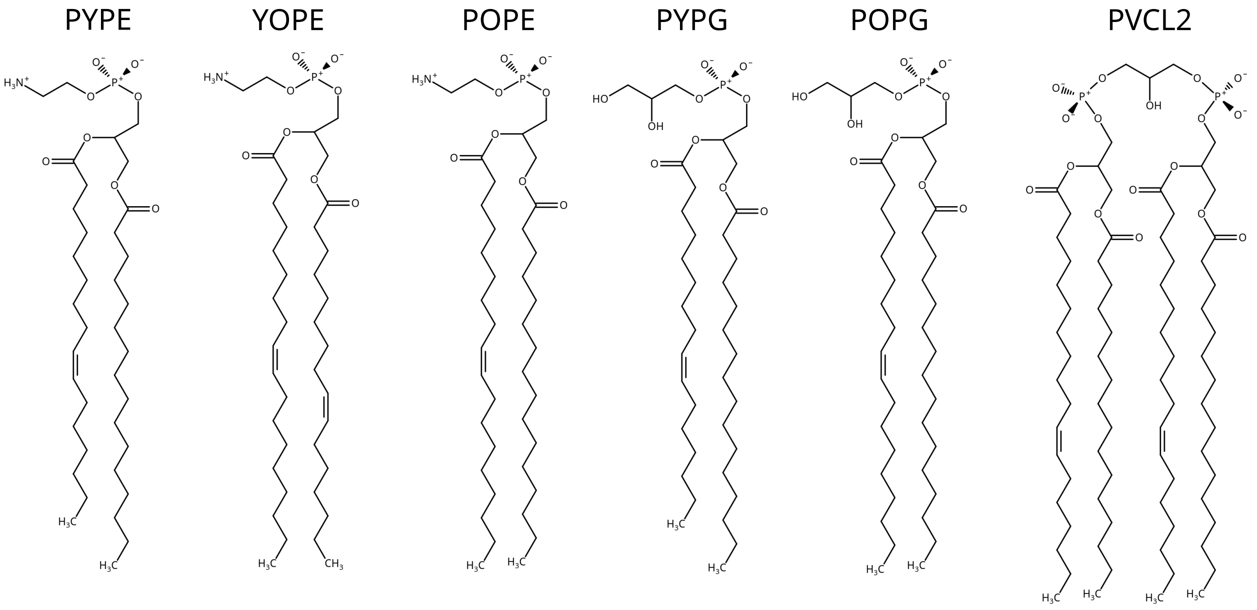

Figure S1: 2D structures of the lipids used in the composition of the lipid membrane. Three Phosphatidylethanolamine (PE) lipids : 1-palmitoyl-2-palmitoleoyl-sn-glycero-3-PE (PYPE) , 1-palmitoyl-2-oleoyl-sn-glycero-3-PE (POPE) and 1-palmitoleoyl-2-oleoyl-glycero-3-PE (YOPE). Two Phosphatidylglycerol (PG) lipids : 1-palmitoyl-2-palmitoleoyl-sn-glycero-3-PG (PYPG) and 1-palmitoyl-2-oleoyl-sn-glycero-3-PG (POPG). One Cardiolipin : 1'[1,2-dipalmitoyl-sn-glycero-3-phospho],3'[1,2-dioleoyl-sn-glycero-3-phospho]-bis glycerol (PVCL2).

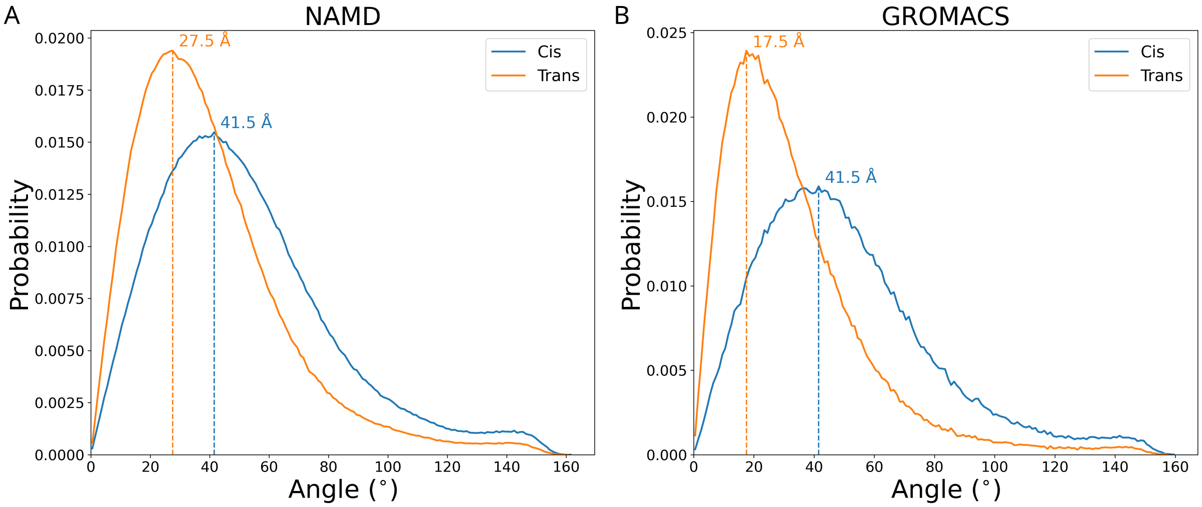

Figure S2: Density distribution of kink angle around the lipid tail unsaturation in (A) NAMD simulations and (B) GROMACS simulations, measured from the carbonyl carbon through the unsaturation site to the terminal carbon on the fatty acid tail. The solid line is the distribution observed over all five replicates, Dashed line is the highest probability of angle observed in different membrane configuration. The text represent the angle that has the highest probability

Table S1: Average diffusion coefficient of lipid residues in the cis and trans membranes calculated by equation 3 over the simulation trajectory, dividing each trajectory into 200ns chunks for independent analysis.

	NAMD		GROMACS	
Lipids	Diffusion Coefficient (10^{-8} cm^2/s) Cis	Trans	Diffusion Coefficient (10^{-8} cm^2/s) Cis	Trans
YOPE	6.8 ± 0.7	5.5 ± 0.3	5.8 ± 0.5	3.1 ± 0.6
PYPE	6.7 ± 0.6	5.2 ± 0.4	6.3 ± 0.6	2.7 ± 0.5
POPE	6.2 ± 0.6	5.2 ± 0.4	4.8 ± 0.7	2.7 ± 0.5
PYPG	7.1 ± 0.6	5.3 ± 0.5	6.0 ± 0.6	3.0 ± 0.7
POPG	6.3 ± 0.8	5.3 ± 0.4	5.4 ± 0.7	2.6 ± 0.4
PVCL2	4.4 ± 0.6	2.6 ± 0.3	2.0 ± 0.3	2.0 ± 0.4

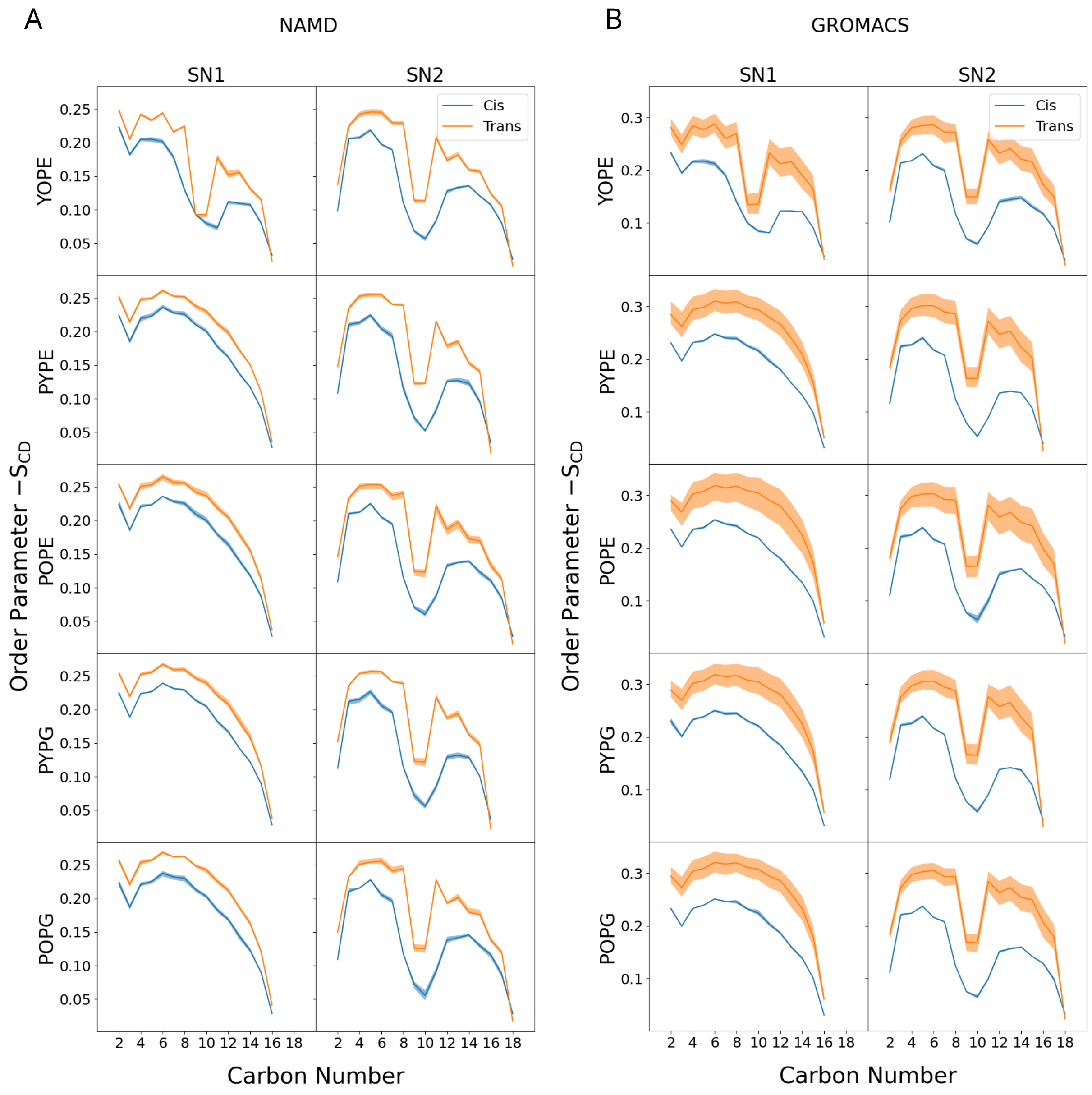

Figure S3: Lipid order parameter calculated for each lipid residue along the different SN1 and SN2 tails using equation 1 in (A) NAMD simulations and (B) GROMACS simulations. The solid lines represent the mean order parameter over all data, while the shaded region accounts for the variation seen over all five simulation replicates.

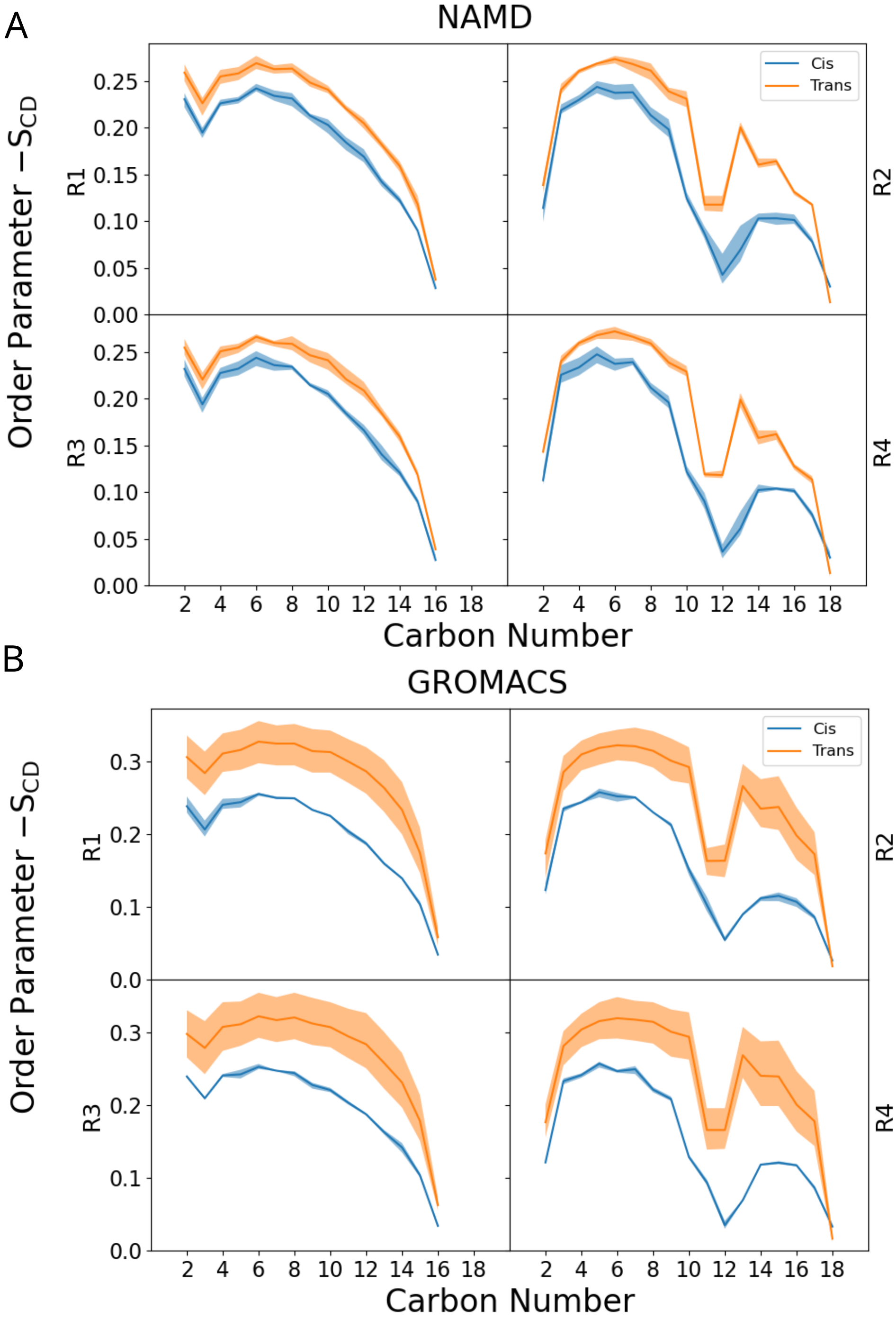

Figure S4: Calculated order parameter for the four cardiolipin tails using equation 1 in (A) NAMD simulations and (B) GROMACS simulations. The solid lines represent the mean order parameter over all data, while the shaded region accounts for the variation seen over all five simulation replicates.
